# AI-related measures and self-reported lesson-aim attainment: an explainable machine-learning analysis of TALIS 2024

**DOI:** 10.3389/fpubh.2026.1911565

**Published:** 2026-08-28

**Authors:** ZhuFang Mao

**Affiliations:** Full-time Education Branch, Jinhua College of Education, Jinhua, China

**Keywords:** artificial intelligence, explainable machine learning, health-promoting schools, LightGBM, self-reported lesson-aim attainment, SHAP, TALIS 2024

## Abstract

**Introduction:**

Artificial intelligence (AI) is increasingly used to support teachers' lesson planning, feedback, assessment, and learning-material adaptation in school settings. Effective teaching practices may also support health literacy, social-emotional development, and health-promoting learning environments, although TALIS does not directly measure public-health education outcomes. Existing studies often treat AI use as a simple adoption variable and provide limited cross-national evidence on whether AI-related measures provide incremental predictive information after accounting for teacher background, teachers' work-related stress, and education-system context.

**Methods:**

A nested explainable machine-learning framework was developed using TALIS 2024 teacher data to test the incremental predictive information supplied by AI-related measures. The participants were 88,991 primary, lower-secondary, and upper-secondary teachers (ISCED 1–3) from 55 countries/economies who were eligible for the AI-module analysis and had a valid outcome. The seven-item outcome captured teachers' reports of how far lessons taught in the previous week achieved specified instructional and classroom-management aims; a relatively high indicator denoted scores at or above the 75th percentile within the same country/economy and ISCED stratum. Matched baseline and AI-added regularized LightGBM models were compared using country-by-school grouped validation and early stopping. Threshold, continuous-outcome, survey-weighted-evaluation, missingness, and country-heterogeneity sensitivity analyses were conducted.

**Results:**

In the final matched analysis, the AI-added model increased independent-test AUROC from 0.6021 to 0.6299 and AUPRC from 0.4138 to 0.4476, while reducing the Brier score from 0.2114 to 0.2071 and log loss from 0.6126 to 0.6029. Paired bootstrap intervals indicated statistically stable, but practically modest, gains (observed ΔAUROC = 0.0277, 95% CI 0.0215–0.0334; observed ΔAUPRC = 0.0338, 95% CI 0.0262–0.0411). AUROC gains were similar at the 70th and 80th percentile thresholds, and the continuous-outcome *R*^2^ increased from 0.1745 to 0.1911. Country-specific AUROC gains were positive in 48 of 55 countries/economies but varied substantially.

**Discussion:**

AI-related perceptions and breadth of task use supplied modest incremental predictive information for predicting relatively high self-reported lesson-aim attainment. “Relatively high” denotes only a within-country/economy and ISCED ranking and does not indicate objectively superior teaching or better student learning. The modest discrimination, cross-sectional design, routed missingness, and self-reported outcome preclude causal interpretation and use for teacher screening or other high-stakes individual decisions. Public-health relevance is presented as a potential extension to future research in health-promoting school settings, not as a directly measured outcome.

## 1 Introduction

Although artificial intelligence (AI) has a long history in education, the public release of ChatGPT on 30 November 2022 accelerated educators' access to generative AI and intensified its use for lesson planning, instructional adaptation, feedback generation, assessment support, and learning analytics (1, 2). This development is primarily educational; its public-health relevance arises when school teaching supports health literacy, social-emotional development, risk awareness, and health-promoting environments. Health-promoting schools are whole-school settings that strengthen healthy living, learning, and working, and recent global standards emphasize integrated systems for health and learning (3, 4). TALIS does not directly measure student health literacy, health behavior, or public-health programme outcomes. Accordingly, this study examines general school teaching practices and treats implications for health-promoting schools as a bounded extension rather than evidence about medical schools, university public-health programmes, or health outcomes.

Recent research has examined AI in teaching from several perspectives. Broad reviews of AI in education have highlighted the shift from AI-directed systems to AI-supported and AI-empowered learning, while also noting limited educator-centered evidence (5–7). Studies of generative AI and large language models further show opportunities for feedback, content generation, and tutoring, alongside risks related to accuracy, academic integrity, equity, and responsible use (1, 2, 8, 9). More teacher-focused reviews have shown that AI can support planning, implementation, assessment, and professional development, while also creating challenges related to teacher readiness, pedagogical integration, ethics, privacy, and equity (10–12). At the policy level, the UNESCO AI Competency Framework for Teachers highlights a human-centered mindset, AI ethics, AI foundations and applications, AI pedagogy, and AI for professional learning (13). Broader health-AI literature also stresses the need for human-centered deployment, robust evaluation, and safety-aware interpretation in health-related domains (14–17). Large-scale evidence is also emerging. TALIS 2024 provides cross-national teacher data and includes items on AI use, AI teaching tasks, perceived benefits, perceived risks, and barriers to AI use (18–20). These developments create an opportunity to assess the incremental predictive information supplied by measured AI-related variables in international teacher data. The present study does not operationalise or validate a distinct construct of “AI-related teacher readiness.”

However, three challenges remain. First, many studies treat AI use as a binary adoption variable, whereas self-reported lesson-aim attainment may be predictively related to perceived benefits, perceived risks, task-use breadth, barriers, stress, workload, and education-system context. Second, international survey data such as TALIS contain questionnaire-specific valid ranges, skip patterns, and special missing codes; if these are not handled using the official codebook logic, machine-learning explanations may be distorted. Third, many educational machine-learning studies report model performance and feature importance but do not explicitly test whether AI-related variables add predictive information beyond baseline teacher and system-level covariates or whether results vary across country contexts.

To address these challenges, this study proposes a codebook-aware nested explainable machine-learning framework. The outcome, relatively high self-reported lesson-aim attainment (SLAA), is constructed from seven TALIS self-report items and defined relative to the same country/economy and ISCED population stratum. The framework then compares a matched baseline model including teacher background, country/economy, and teachers' work-related stress with an AI-added model that includes perceived AI benefits, perceived AI risks, breadth of AI task use, and AI-use barriers. LightGBM is used as the primary model because gradient-boosted decision trees are effective for mixed tabular data (21), and SHAP is used for interpretable feature attribution (22). Incremental predictive information is evaluated using country-by-school group validation, paired bootstrap confidence intervals, calibration analysis, alternative outcome thresholds, a continuous outcome, survey-weighted test metrics, and country-specific analyses.

**Figure 1:**
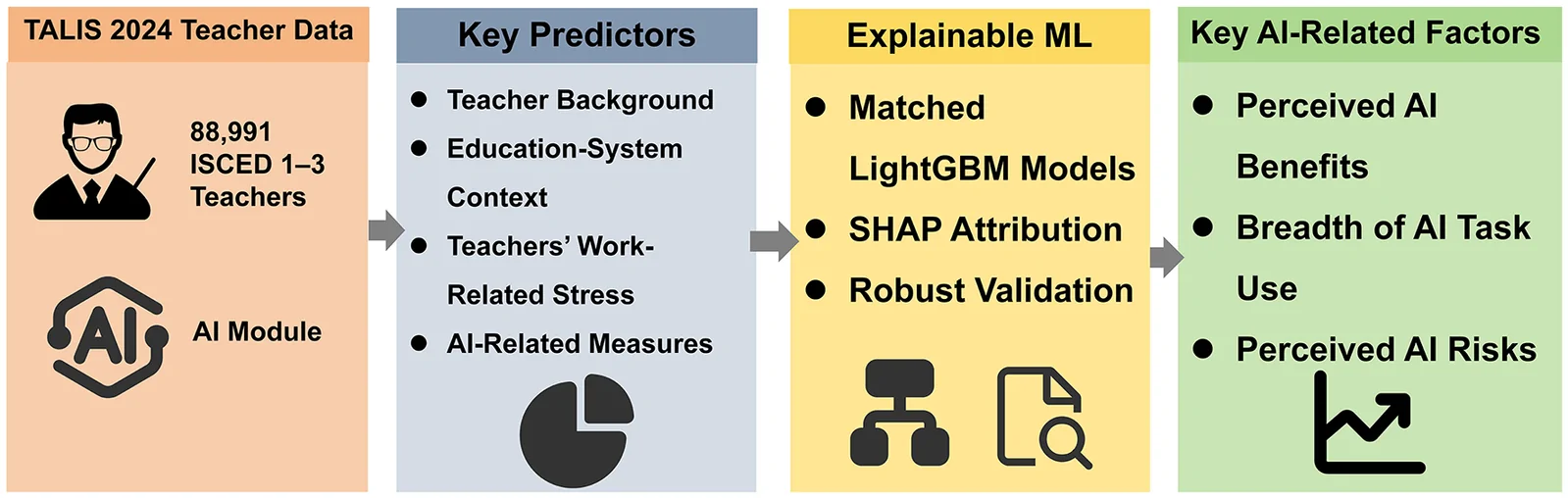
Conceptual Overview of AI-Related Measures and Self-Reported Lesson-Aim Attainment. TALIS 2024 teacher data were used to examine how teacher background, education-system context, the broad work-related stress index, and AI-related measures contribute to predictions of relatively high SLAA. The arrows indicate predictive relationships rather than causal effects, and any public-health relevance is an indirect extension for future research in health-promoting school settings.

The main contributions of this study are threefold. First, it constructs a codebook-aware TALIS 2024 pipeline for measured AI-related variables and SLAA, explicitly accounting for valid response ranges, special missing codes, skip-pattern variables, and a within-CNTRY×IDPOP outcome definition. Second, it introduces a matched nested modeling strategy that quantifies the incremental predictive information supplied by AI-related predictors beyond background, country/economy, and teachers' work-related stress, rather than merely ranking variables in a single full model. Third, it provides a multi-layer validation design using country-by-school group splits, paired bootstrap confidence intervals, calibration, alternative thresholds, a continuous outcome, survey-weighted test metrics, and country-specific heterogeneity analyses. Figure 1 summarizes the conceptual framework.

The remainder of this article is organized as follows. Section 2 reviews related work on AI-supported teaching, health-oriented education, and explainable tabular machine learning. Section 3 describes the data source, outcome construction, codebook-aware cleaning, modeling framework, and validation procedures. Section 4 reports the experimental results, including SOTA comparison, ablation, robustness, stability, calibration, and visual explanations. Section 5 discusses the findings and limitations. Section 6 concludes the study.

## 2 Related work

### 2.1 AI-supported teaching practices and health-promoting school settings

Prior studies relevant to AI-supported school teaching and possible extensions to health-promoting school research can be grouped into four streams. The first stream concerns school-based health education and health-promoting schools. The World Health Organization conceptualizes health-promoting schools as whole-school settings that strengthen their capacity for healthy living, learning, and working; global WHO–UNESCO standards further emphasize whole-school systems for health and learning (3, 4). This literature establishes a possible setting for future research, but it does not make the general TALIS teacher sample representative of public-health education. The second stream examines AI in teaching and teacher professional development. Earlier and recent reviews show that AI may support planning, implementation, assessment, and professional learning while raising challenges related to teacher readiness, pedagogical integration, data privacy, and ethical use (5, 10–12). The third stream studies technology acceptance, AI competence, and responsible AI. Classical acceptance models emphasized perceived usefulness and ease of use (23, 24); more recent education-specific work emphasizes AI paradigms, ethical principles, generative-AI guidance, and teacher competencies (1, 7, 9, 13). The fourth stream uses large-scale teacher surveys to study teaching conditions, professional learning, stress, and classroom practices. TALIS 2024 is especially relevant because it provides cross-national teacher and school-leader data and includes an AI module covering use, tasks, benefits, risks, and non-use reasons (18–20, 25).

Different from prior studies, the present work does not treat AI use as a single adoption variable or evaluate one local AI intervention. Instead, it uses TALIS 2024 to construct a codebook-cleaned, cross-national teacher-level analysis of relatively high SLAA. The study further connects AI-related benefits, risks, breadth of task use, and barriers with teachers' work-related stress and background factors, and includes a health/physical-education-related subset as a same-source analytic scenario rather than external validation.

### 2.2 Explainable and validated tabular machine learning for predictive-information assessment

The methodological literature related to this study can be grouped into three categories. The first category is predictive modeling for structured tabular data. Logistic and penalized regression remain useful transparent baselines, while ensemble tree methods often perform strongly on mixed survey-type data. Random forests, gradient boosting, XGBoost, LightGBM, and CatBoost are established representatives of this family (21, 26–29). Recent comparative work also suggests that tree-based models remain difficult to outperform on many typical tabular datasets (30). In the past two years, tabular deep learning and tabular foundation models have developed rapidly, including TabR, TabM, TabPFN, TabICL, RealMLP, and Mambular (31–36). The second category is explainable AI for education. SHAP provides a theoretically grounded additive feature-attribution framework, and subsequent tree-SHAP work demonstrated how local explanations can be combined into global model understanding (22, 37). Education-focused XAI work further emphasizes that interpretability should be tied to stakeholder trust, pedagogical utility, and validation of explanations (38). The third category is validation design for educational machine learning. Existing educational-data-mining studies often report discrimination metrics and feature importance, but fewer studies examine whether variable importance remains stable after model refitting, whether variable blocks add information beyond baseline covariates, or whether results transport across country contexts.

This study differs from the above methodological work by integrating these elements into a single predictive-information assessment framework rather than proposing a new classifier alone. Specifically, it combines codebook-aware TALIS cleaning, a within-CNTRY×IDPOP outcome definition, matched nested LightGBM ablation, descriptive algorithm benchmarking, SHAP interpretation, paired bootstrap confidence intervals, country-by-school group validation, calibration analysis, outcome-definition sensitivity analyses, survey-weighted evaluation, and country-specific heterogeneity analysis. Each step addresses a concrete source of bias or uncertainty when assessing AI-related predictive information for SLAA.

## 3 Materials and methods

### 3.1 Study design and data source

This secondary cross-sectional study used the TALIS 2024 teacher public-use file. TALIS employs stratified two-stage probability sampling: schools are sampled within explicitly and implicitly defined strata, followed by random sampling of eligible teachers within participating schools; participation and response-rate procedures are documented by the OECD (18, 25). Participants were primary, lower-secondary, and upper-secondary teachers in the ISCED 1, 2, and 3 populations. The primary objective was conditional prediction in the assembled analytic sample, so models were fitted without final teacher weights and are not design-based population estimates. As a sensitivity analysis, all test metrics were recalculated using the final teacher weight. Country/economy and school identifiers were concatenated to form 16,799 unique school groups, preventing schools from appearing in both training and test data. Country/economy was included as context, and country-specific performance was examined. Survey clustering, unequal selection probabilities, and system-specific response patterns remain limitations.

### 3.2 Outcome construction

The outcome is termed *self-reported lesson-aim attainment* (SLAA). TALIS item 58 asks teachers: “To what extent have the lessons you taught over the past week in the target class achieved the following aims?” The seven aims are presenting content comprehensibly, engaging students in challenging work, providing feedback, offering practice opportunities, adapting teaching to student needs, supporting students' emotional and behavioral self-management, and managing student behavior (19). Thus, the measure is a recent, class-specific teacher report; it is not an objective or comprehensive assessment based on student outcomes, observation, or supervisor evaluation. The SLAA score was the mean of at least four valid items. In the final analytic sample, internal consistency was good (Cronbach's α = 0.866, McDonald's one-factor ω = 0.867, complete-item *n* = 86, 617; mean communality 0.483). These results support aggregation for prediction but do not establish criterion validity or cross-national measurement invariance. Let *x*_*ij*_ denote the cleaned response of teacher *i* to item *j*∈{1, …, 7}, and let *m*_*i*_ be the number of valid responses. The continuous score was defined as


$$\begin{array}{l} LAA_{i}=\frac{1}{m_{i}}\overset{7}{\underset{j=1}{\sum }}x_{ij},\;m_{i}\geq 4. \end{array}$$ (1)

For each stratum *g* = (CNTRY, IDPOP), the binary outcome was


$$\begin{array}{l} y_{i}=𝕀\left(LAA_{i}\geq Q_{0.75}\left(LAA|g_{i}\right)\right), \end{array}$$ (2)

where *Q*_0.75_(*LAA*∣*g*_*i*_) is the 75th percentile among teachers in teacher *i*'s country/economy and ISCED stratum. The 75th percentile was selected a priori as a transparent upper-quartile operationalization that retained sufficient positive cases for grouped modeling; it is not a validated absolute cut-point. The label therefore means *relatively high SLAA within the same national and education-level context*. It does not denote objectively superior teaching quality or demonstrate better student learning outcomes. Dichotomisation loses within-category information, and ties yielded a 32.1% positive rate in the independent test set rather than exactly 25%. The full matched analysis was therefore repeated at the 70th and 80th percentiles, and a continuous-outcome model was fitted.

### 3.3 AI-related predictors and non-AI covariates

AI-related predictors were constructed from the TALIS 2024 AI module (19). In the final analytic sample, perceived AI benefits had α = 0.907 and ω = 0.909 (complete-item *n* = 55, 610), perceived AI risks had α = 0.841 and ω = 0.845 (*n* = 43, 067), and the 20-item broad work-related stress index had α = 0.913 and ω = 0.913 (*n* = 81, 584). One-factor mean communalities were 0.668, 0.524, and 0.350, respectively. For the stress index, the high coefficients may partly reflect the number of items, whereas the low mean communality does not support a strong unidimensional interpretation. The index was retained only as a broad baseline adjustment variable covering multiple sources and experiences of work-related stress; it was not treated as a validated single latent stress construct or interpreted as a focal substantive exposure. Full convergent/discriminant validity and cross-national invariance were not established. For teacher *i*, the AI perception scores were defined as shown in Equation 3.


$$\begin{array}{l} B_{i}=\frac{1}{\left|B_{i}\right|}\underset{j\in B_{i}}{\sum }b_{ij},\;R_{i}=\frac{1}{\left|R_{i}\right|}\underset{j\in R_{i}}{\sum }r_{ij}, \end{array}$$ (3)

where *b*_*ij*_ and *r*_*ij*_ are cleaned responses on the valid 1–4 scale. Breadth of AI task use was defined as the number of endorsed AI-supported teaching-task types across nine binary items TT4G37A–TT4G37I. Breadth of AI task use was calculated as shown in Equation 4.


$$\begin{array}{l} T_{i}=\overset{9}{\underset{j=1}{\sum }}z_{ij},\;z_{ij}\in \left\{0,1\right\}. \end{array}$$ (4)

This count is a formative index: its nine task types need not be interchangeable manifestations of one latent construct, so internal-consistency coefficients are inappropriate. It measures variety, not frequency, quality, depth, competence, or ethical appropriateness of AI integration. AI-use barriers were represented by binary items TT4G38A–TT4G38F. Non-AI covariates included teacher background, employment/workload indicators, country/economy, ISCED stratum, and the broad work-related stress index. The stress index used TT4G76A–TT4G76D and TT4G77A–TT4G77P, with TT4G76B reverse-coded.

### 3.4 Codebook-aware cleaning

All variables were cleaned using item-specific valid ranges. AI task and barrier items were recoded as 1 = Yes↦1 and 2 = No↦0; applicable special codes were set to missing. AI benefit, risk, stress, and outcome items were retained only on their valid 1–4 scales. The raw file contained 278,383 teachers. A valid AI-use routing response identified 89,833 AI-eligible records (32.3%); 842 additional records lacked a valid outcome or grouping field, leaving 88,991 teachers. The corresponding exclusions were 188,550 and 842 records. In the final sample, missingness was 21.1% for AI benefits, 27.1% for AI risks, 59.1% for task-use breadth, and 0.9% for stress. Numerical predictors were median-imputed with missingness indicators and categorical predictors were mode-imputed and one-hot encoded; all preprocessing parameters were learned in training data only. Outcomes were never imputed. Multiple imputation was not used because most task-breadth absence followed questionnaire routing rather than ordinary item nonresponse. Consequently, the model can use both the observed breadth values and pathway information carried by the breadth missingness indicator. Source-level attribution for this variable cannot be interpreted as attribution to actual breadth of AI use alone. Comparisons of six available background indicators between AI-eligible and non-eligible records produced absolute standardized mean differences of 0.055 or less, but unmeasured selection remains possible. Sample-selection flow and missingness are summarized in Table 1, and reliability and one-factor evidence for the reflective composite measures are reported in Table 2. The complete codebook-aware cleaning and outcome-construction procedure is summarized in Algorithm 1.

**Table 1:** Sample selection flow and principal sources of missingness.

Stage or focal measure	Retained *n*	Excluded or missing %
Raw TALIS 2024 teacher records	278,383	–
Valid AI-module routing response	89,833	188,550 excluded
Valid SLAA, country/economy, ISCED, and school identifier	88,991	842 excluded
Perceived AI benefits in final sample	70,225	21.1% missing
Perceived AI risks in final sample	64,909	27.1% missing
Breadth of AI task use in final sample	36,435	59.1% missing
Teachers' work-related stress in final sample	88,195	0.9% missing

**Table 2:** Reliability and one-factor evidence for reflective composite measures.

Measure	Items	Complete *n*	α	ω/communality
Self-reported lesson-aim attainment	7	86,617	0.866	0.867/0.483
Perceived AI benefits	5	55,610	0.907	0.909/0.668
Perceived AI risks	5	43,067	0.841	0.845/0.524
Teachers' work-related stress	20	81,584	0.913	0.913/0.350

α, Cronbach's alpha; ω, McDonald's one-factor omega. Communality is the mean one-factor communality. The breadth-of-task-use count is formative and is therefore not included.

Algorithm 1 Codebook-aware cleaning and outcome construction.

**Input:** TALIS 2024 teacher file; variable-specific valid ranges; AI module variables; outcome items TT4G58A–TT4G58G.
**Step 1:** Recode AI task and AI barrier items as binary variables: Yes = 1, No = 0, special and invalid codes = missing.
**Step 2:** Restrict AI benefit/risk, stress, and lesson-aim-attainment items to their valid response ranges.
**Step 3:** Reverse-code TT4G76B and construct the stress score.
**Step 4:** Construct *LAA*_*i*_ using Equation 1; require at least four valid TT4G58 responses.
**Step 5:** Within each CNTRY×IDPOP group, calculate the 75th percentile of *LAA*_*i*_ and construct *y*_*i*_ using Equation 2.
**Output:** Codebook-cleaned analytic data with a binary relatively high SLAA label.

### 3.5 Matched nested LightGBM model

The primary model was LightGBM, a gradient-boosted decision-tree method building on gradient boosting principles and designed for efficient modeling of large structured datasets (21, 27). To test whether AI variables provide incremental information beyond baseline covariates, two matched nested models were fitted. The matched baseline and AI-added models are defined in Equations 5 and 6, respectively.


$$\begin{array}{ll} \text{Model 1:}{\hat{p}}_{i}^{\left(1\right)} & =f_{1}\left({\text{background}}_{i},{\text{country/economy}}_{i},{\text{stress}}_{i}\right), \end{array}$$ (5)

$$\begin{array}{ll} \text{Model 2:}{\hat{p}}_{i}^{\left(2\right)} & =f_{2}\left({\text{background}}_{i},{\text{country/economy}}_{i},{\text{stress}}_{i},{\text{AI}}_{i}\right), \end{array}$$ (6)

where ${\hat{p}}_{i}$ is the predicted probability of relatively high SLAA. Model 1 was the non-AI baseline, and Model 2 was the AI-added model.

Both final models used identical country-by-school outer and inner grouped splits. The outer split used 70,864 teachers for training and 18,127 for independent testing. Model 1 used 22 raw predictors and Model 2 used 31. The selected configuration used learning rate 0.025, 15 leaves, maximum depth 5, minimum child size 100, row and column subsampling 0.75, L1 regularization 0.1, and L2 regularization 5.0. Early stopping selected 529 and 546 iterations for Models 1 and 2, respectively.

**Figure 2:**
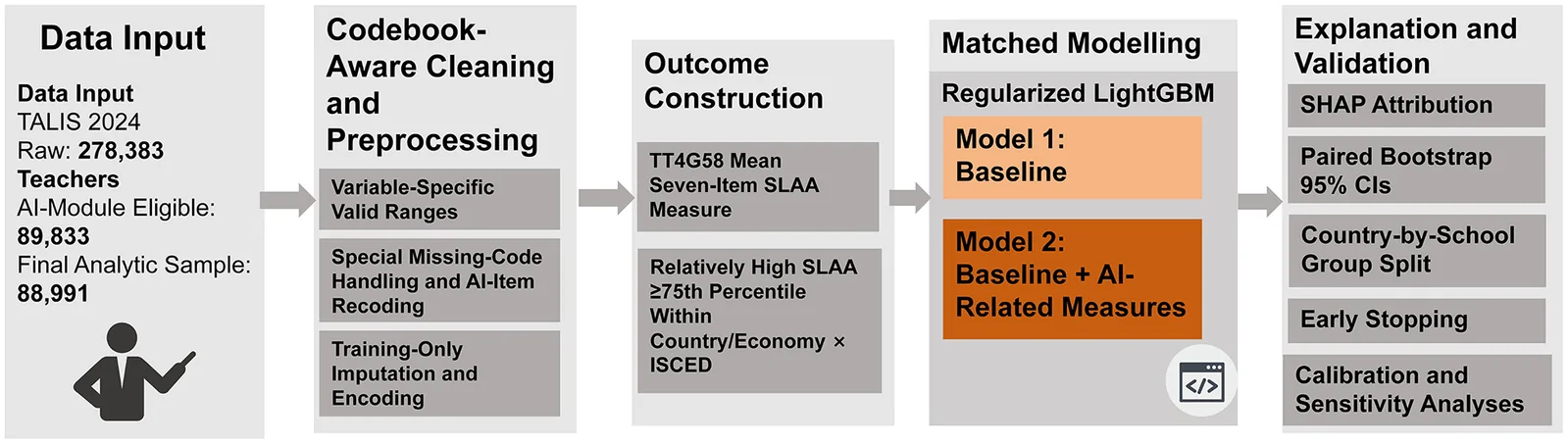
Codebook-aware nested explainable machine-learning pipeline. TALIS 2024 teacher data were cleaned using variable-specific valid ranges, special missing-code handling, and AI-item recoding. SLAA was constructed from the TT4G58 items, and the relative-high indicator was defined within CNTRY×IDPOP strata. Matched baseline and AI-added regularized LightGBM models were compared using country-by-school group validation, early stopping, paired bootstrap confidence intervals, calibration, sensitivity analyses, and SHAP-based interpretation.

Figure 2 presents the full codebook-aware nested modeling pipeline used in the final analysis. Model performance was evaluated by AUROC, AUPRC, Brier score, and log loss. Brier score and calibration curves were included because discrimination alone is insufficient for judging probability quality (39, 40). For an evaluation metric *M*, incremental predictive information was defined as shown in Equation 7.


$$\begin{array}{l} \text{Δ}M=M\left({\hat{p}}^{\left(2\right)},y\right)-M\left({\hat{p}}^{\left(1\right)},y\right), \end{array}$$ (7)

where positive values indicate improvement for AUROC and AUPRC, while negative values indicate improvement for Brier score and log loss. Paired bootstrap confidence intervals were calculated by resampling test-set observations with replacement 500 times and recomputing Δ*M* for each bootstrap sample (41). The complete matched nested modeling and validation workflow is summarized in Algorithm 2.

Algorithm 2 Codebook-aware matched nested explainable machine-learning framework. 
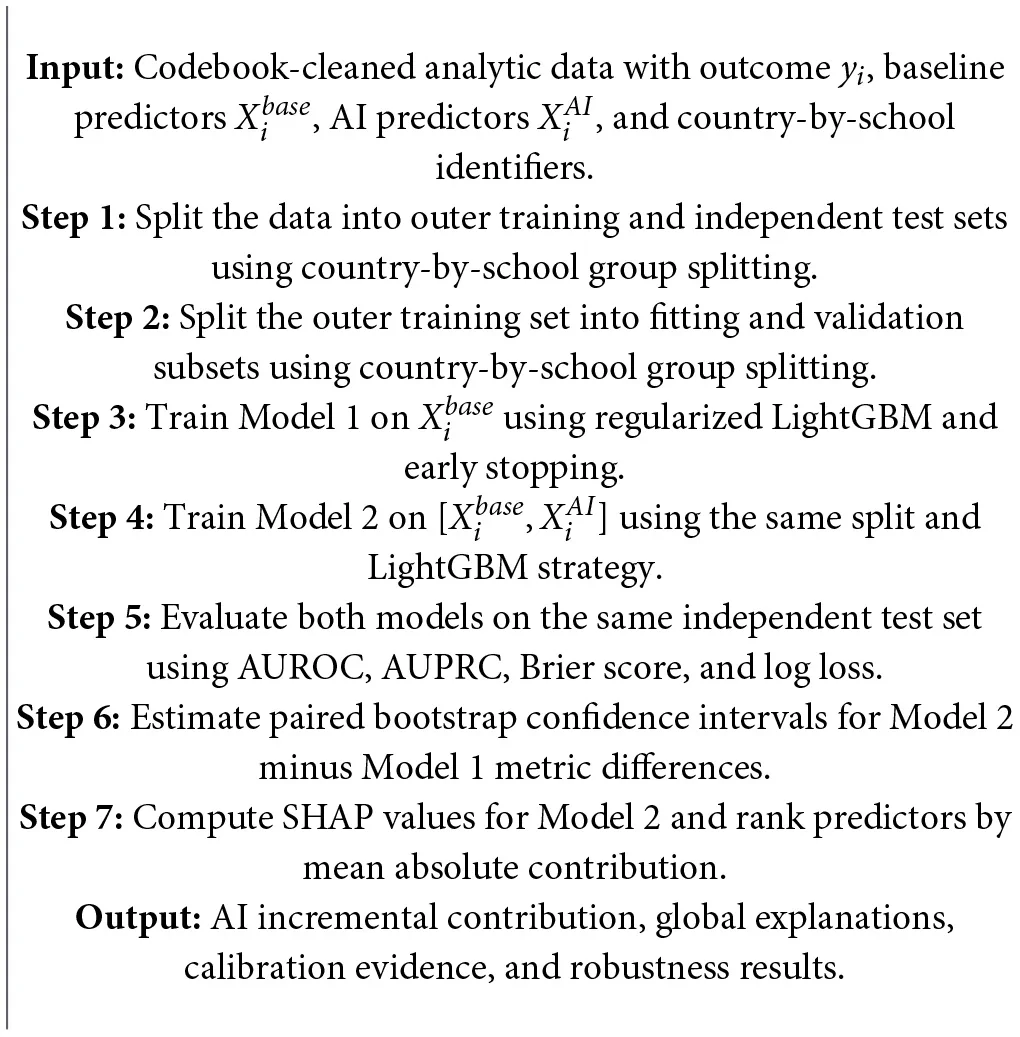

### 3.6 Explainability

SHAP values were used to explain Model 2, following the SHAP additive explanation framework and tree-based SHAP interpretation literature (22, 37). The SHAP additive decomposition and global feature importance were calculated using Equations 8 and 9, respectively. For a feature vector *x*_*i*_, the SHAP additive explanation can be written as


$$\begin{array}{l} g\left(x_{i}\right)=\phi_{0}+\overset{K}{\underset{k=1}{\sum }}\phi_{ik}, \end{array}$$ (8)

where ϕ_*ik*_ is the contribution of feature *k* to the prediction for teacher *i*. Global feature importance was calculated as


$$\begin{array}{l} I_{k}=\frac{1}{n}\overset{n}{\underset{i=1}{\sum }}|\phi_{ik}|. \end{array}$$ (9)

SHAP importance is predictive attribution conditional on the fitted model and feature set. It is not a regression coefficient, statistical significance test, causal effect, or measure of intervention actionability. Correlated predictors can share or redistribute attribution; consequently, rankings were interpreted as descriptions of model behavior and not as stand-alone policy priorities. SHAP values were calculated for a fixed random sample of 8,000 independent-test observations and aggregated from encoded columns to their source variables. For task breadth, this source-level aggregation includes the encoded breadth value and its routing-related missingness indicator; the resulting importance is therefore not a pure measure of breadth of AI use. Interpretive considerations were evaluated against educational theory, existing evidence, and the limitations of the cross-sectional design, without treating SHAP ranks as intervention recommendations.

## 4 Experiments and results

### 4.1 Experimental datasets and settings

The two analytic scenarios and their sample sizes are summarized in Table 3. Two TALIS 2024 analytic scenarios were used. Dataset A was the main TALIS-AI sample. Dataset B comprised teachers identified by either the single-subject code for physical education/health (TT4G48A = 9) or endorsement of physical education/health in the multi-subject item (TT4G48BI = 1). It therefore mainly represents school physical-education/health teaching and is not representative of public-health education, medical education, or university-level public-health programmes. The subset offers a closer, but still indirect, link to health-promoting school practice; TALIS contains no direct public-health curriculum or student health-outcome measure. Both scenarios came from the same TALIS source, so Dataset B is a descriptive sensitivity scenario rather than independent or external validation.

**Table 3:** Summary of the two TALIS 2024 analytic scenarios used for common-split algorithm comparison.

Dataset	Raw rows	Model rows	Train rows	Test rows	Features	Countries
Dataset A: TALIS-AI	89,833	88,991	70,864	18,127	44	55
Dataset B: Health/PE subset	21,938	21,687	17,243	4,444	44	51

All compared algorithms used the same 75th-percentile outcome, 44 source predictors, preprocessing learned from training data only, and independent test partition defined by the concatenated country/economy and school identifier. The compared methods were L2-penalized logistic regression, random forest, extra-trees, LightGBM, XGBoost, and CatBoost. Implementations used scikit-learn-compatible workflows (42). AUROC and AUPRC assessed discrimination and class retrieval, while Brier score and log loss assessed probability quality. Hyperparameter budgets were not exhaustively equalized, so the comparison is descriptive rather than evidence of universal algorithm superiority.

### 4.2 Common-split algorithm comparison

**Figure 3:**
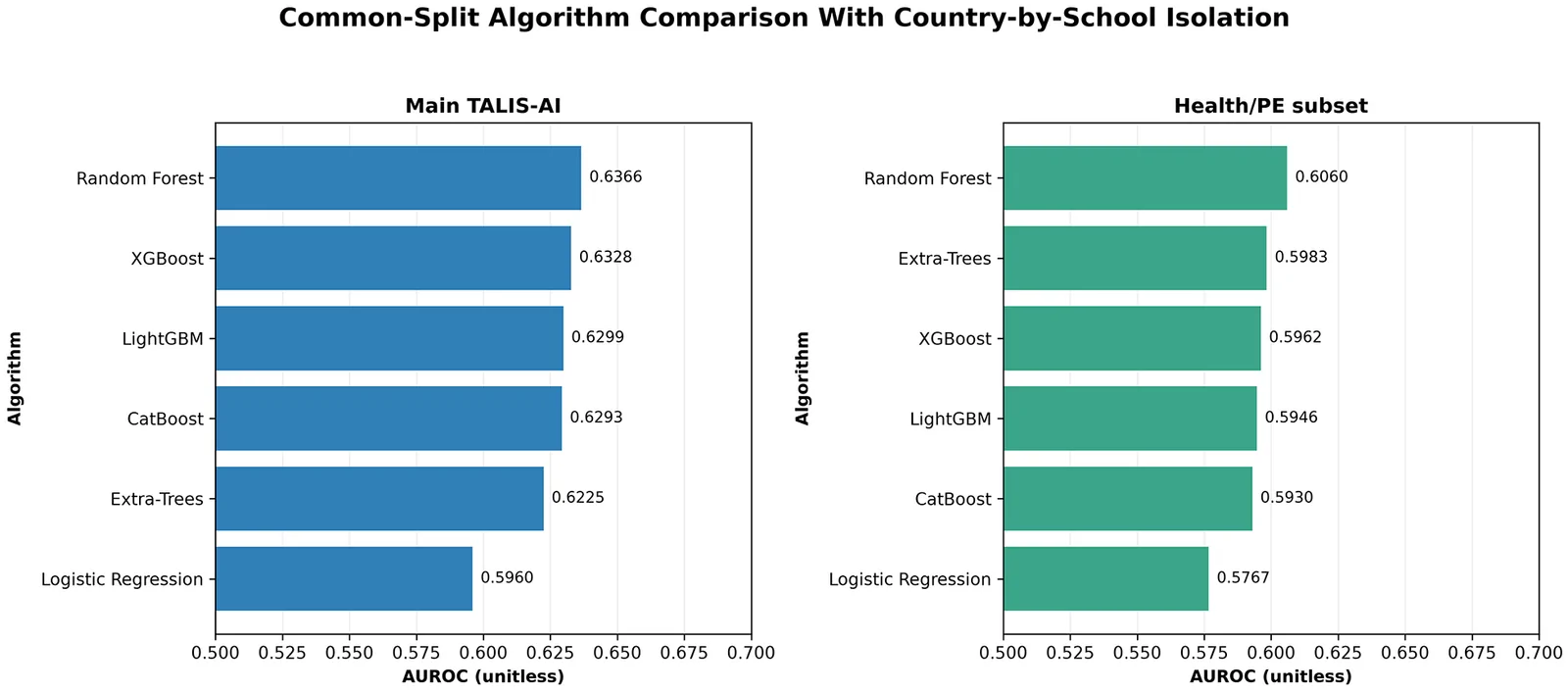
Common-split algorithm comparison on two TALIS 2024 analytic scenarios. Both panels use the same country-by-school isolation rule and training-only preprocessing; **(A)** shows the main TALIS-AI scenario and **(B)** the Health/PE subset.

Table 4 and Figure 3 report the corrected common-split comparison. On Dataset A, random forest had the highest AUROC (0.6366) and AUPRC (0.4563), whereas XGBoost had the lowest Brier score (0.2067) and log loss (0.6019). On Dataset B, random forest again had the highest AUROC (0.6060) and AUPRC (0.4364), while XGBoost had the lowest Brier score (0.2124) and log loss (0.6154). The Health/PE results no longer suggest unusually high discrimination after country-by-school isolation; they support the same cautious interpretation as the main scenario.

**Table 4:** Common-split algorithm comparison on two TALIS 2024 analytic scenarios. Best results within each scenario are shown in bold.

Dataset	Method	AUROC ↑	AUPRC ↑	Brier ↓	Log loss ↓
Dataset A: TALIS-AI	Random forest	**0.6366**	**0.4563**	0.2243	0.6407
	XGBoost	0.6328	0.4498	**0.2067**	**0.6019**
	LightGBM	0.6299	0.4476	0.2071	0.6029
	CatBoost	0.6293	0.4482	0.2073	0.6035
	Extra-trees	0.6225	0.4363	0.2338	0.6602
	Logistic regression (L2)	0.5960	0.4058	0.2428	0.6787
Dataset B: Health/PE subset	Random forest	**0.6060**	**0.4364**	0.2281	0.6486
	Extra-trees	0.5983	0.4180	0.2360	0.6649
	XGBoost	0.5962	0.4249	**0.2124**	**0.6154**
	LightGBM	0.5946	0.4235	0.2127	0.6162
	CatBoost	0.5930	0.4219	0.2125	0.6155
	Logistic regression (L2)	0.5767	0.3913	0.2464	0.6862

Bold values indicate the best performance within each dataset/scenario; higher values are better for AUROC and AUPRC, whereas lower values are better for Brier score and log loss.

The comparison used the same corrected country-by-school grouped split as the confirmatory analysis. It was used descriptively to assess algorithm-family sensitivity, not to select the scientific estimand or to support the incremental AI-block claim. The confirmatory result remains the matched LightGBM contrast in which the only planned difference between models is addition of the AI-related predictor block.

### 4.3 Final matched ablation: incremental value of AI variables

Independent-test performance for the final matched models is reported in Table 5. The final confirmatory analysis compared two matched regularized LightGBM models. Final Model 1 included teacher background, country/economy, and the broad work-related stress index; Final Model 2 added AI-related predictors. The statistically stable increments were small in absolute terms, and the final AUROC of 0.6299 indicates limited discrimination. The models are therefore appropriate only for exploratory assessment of incremental predictive information at sample or group level, not teacher screening, evaluation, classification, or other high-stakes individual decisions.

**Table 5:** Final matched nested LightGBM performance on the independent test set.

Model	Best iteration	AUROC	AUPRC	Brier	Log loss
Final Model 1: background + country + stress	529	0.6021	0.4138	0.2114	0.6126
Final Model 2: Model 1 + AI	546	**0.6299**	**0.4476**	**0.2071**	**0.6029**

Bold values indicate the better-performing model for each metric; higher values are better for AUROC and AUPRC, whereas lower values are better for Brier score and log loss.

**Figure 4:**
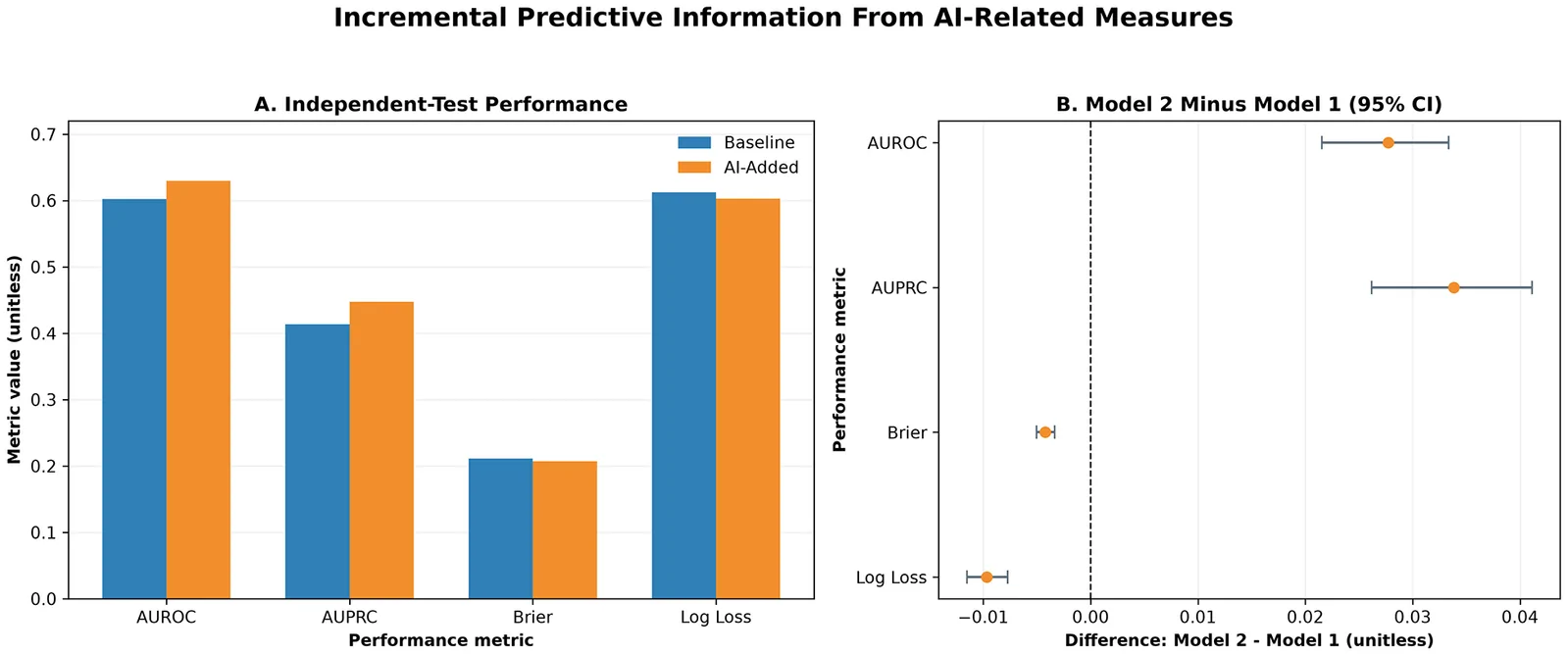
Incremental predictive information from AI-related measures. **(A)** Compares the independent-test performance of the matched baseline and AI-added models. **(B)** Shows paired bootstrap confidence intervals for Model 2 minus Model 1. Positive differences indicate improvement for AUROC and AUPRC; negative differences indicate improvement for Brier score and log loss because lower values are better.

Paired bootstrap analysis showed statistically stable incremental predictive information from AI-related measures (Table 6, Figure 4). Point estimates are the observed test-set differences calculated from the unrounded metrics; bootstrap resampling supplies the confidence intervals. The observed AUROC difference was 0.0277 (95% CI 0.0215–0.0334), and the observed AUPRC difference was 0.0338 (95% CI 0.0262–0.0411). The Brier score and log loss also decreased, with confidence intervals fully below zero.

**Table 6:** Paired bootstrap 95% confidence intervals for incremental predictive information from AI-related measures.

Metric	Observed Model 2–Model 1	95% CI lower	95% CI upper
AUROC	+0.0277	0.0215	0.0334
AUPRC	+0.0338	0.0262	0.0411
Brier	–0.0042	–0.0050	–0.0034
Log loss	–0.0097	–0.0115	–0.0077

### 4.4 Outcome, weighting, and country sensitivity analyses

The direction of the AI-added differences was similar under alternative relative-high thresholds (Table 7). Differences were calculated from unrounded paired predictions and rounded once for reporting. At the 70th, 75th, and 80th percentiles, the observed unweighted AUROC differences were 0.0280, 0.0277, and 0.0255, respectively; the corresponding AUPRC differences were 0.0322, 0.0338, and 0.0299. In the continuous-outcome analysis, adding the AI block increased independent-test *R*^2^ from 0.1745 to 0.1911 and reduced RMSE from 0.4942 to 0.4892. Applying final teacher weights at evaluation, while leaving model fitting unchanged, increased the 75th-percentile AUROC from 0.5878 to 0.6345 (difference 0.0466) and AUPRC from 0.3722 to 0.4281 (difference 0.0559). The lower weighted baseline AUROC and larger weighted increment, compared with the unweighted values of 0.6021, 0.6299, and 0.0277, show that performance magnitude is sensitive to survey weights, country composition, or unequal selection probabilities. These weighted figures are evaluation-only sensitivity estimates rather than fully design-based model fits.

**Table 7:** Outcome-definition and survey-weight sensitivity analyses.

Analysis	Model	AUROC or *R*^2^	AUPRC or RMSE	Brier or MAE	Log loss
70th percentile, unweighted	Baseline	0.5948	0.4630	0.2285	0.6489
70th percentile, unweighted	AI-added	**0.6228**	**0.4951**	**0.2240**	**0.6391**
75th percentile, unweighted	Baseline	0.6021	0.4138	0.2114	0.6126
75th percentile, unweighted	AI-added	**0.6299**	**0.4476**	**0.2071**	**0.6029**
80th percentile, unweighted	Baseline	0.6154	0.3824	0.1907	0.5675
80th percentile, unweighted	AI-added	**0.6409**	**0.4123**	**0.1872**	**0.5589**
75th percentile, weighted evaluation	Baseline	0.5878	0.3722	0.2038	0.5966
75th percentile, weighted evaluation	AI-added	**0.6345**	**0.4281**	**0.1975**	**0.5818**
Continuous SLAA	Baseline	0.1745	0.4942	0.3921	–
Continuous SLAA	AI-added	**0.1911**	**0.4892**	**0.3877**	–

Bold values indicate better performance within each paired analysis (AI-added versus baseline); higher values are better for AUROC, *R*^2^, and AUPRC, whereas lower values are better for RMSE, Brier score, MAE, and log loss.

Country-specific test results showed meaningful heterogeneity. The AUROC difference was positive in 48 of 55 countries/economies, with a median of 0.0281, interquartile range 0.0115–0.0431, and range −0.0432 to 0.1164. The AUPRC difference was positive in 43 of 55 systems. Unadjusted within-country Spearman correlations with continuous SLAA were positive in 46 of 55 systems for perceived AI benefits and 53 of 55 for task-use breadth, but only 27 of 55 for perceived AI risks. Median correlations were 0.0605, 0.0941, and −0.0068, respectively. These descriptive correlations do not adjust for covariates and are reported only to assess cross-context direction, not causality. Country/economy heterogeneity across the 55 independent-test subsets is summarized in Table 8.

**Table 8:** Country/economy heterogeneity across 55 independent-test subsets.

Quantity	Positive systems	Minimum	Q1	Median	Maximum
ΔAUROC	48/55	–0.0432	0.0115	0.0281	0.1164
ΔAUPRC	43/55	–0.0615	0.0044	0.0260	0.1785
Benefits–SLAA Spearman ρ	46/55	–0.0632	0.0198	0.0605	0.2257
Risks–SLAA Spearman ρ	27/55	–0.1572	–0.0347	–0.0068	0.1076
Task breadth–SLAA Spearman ρ	53/55	–0.0537	0.0626	0.0941	0.2728

### 4.5 Calibration and SHAP interpretation

**Figure 5:**
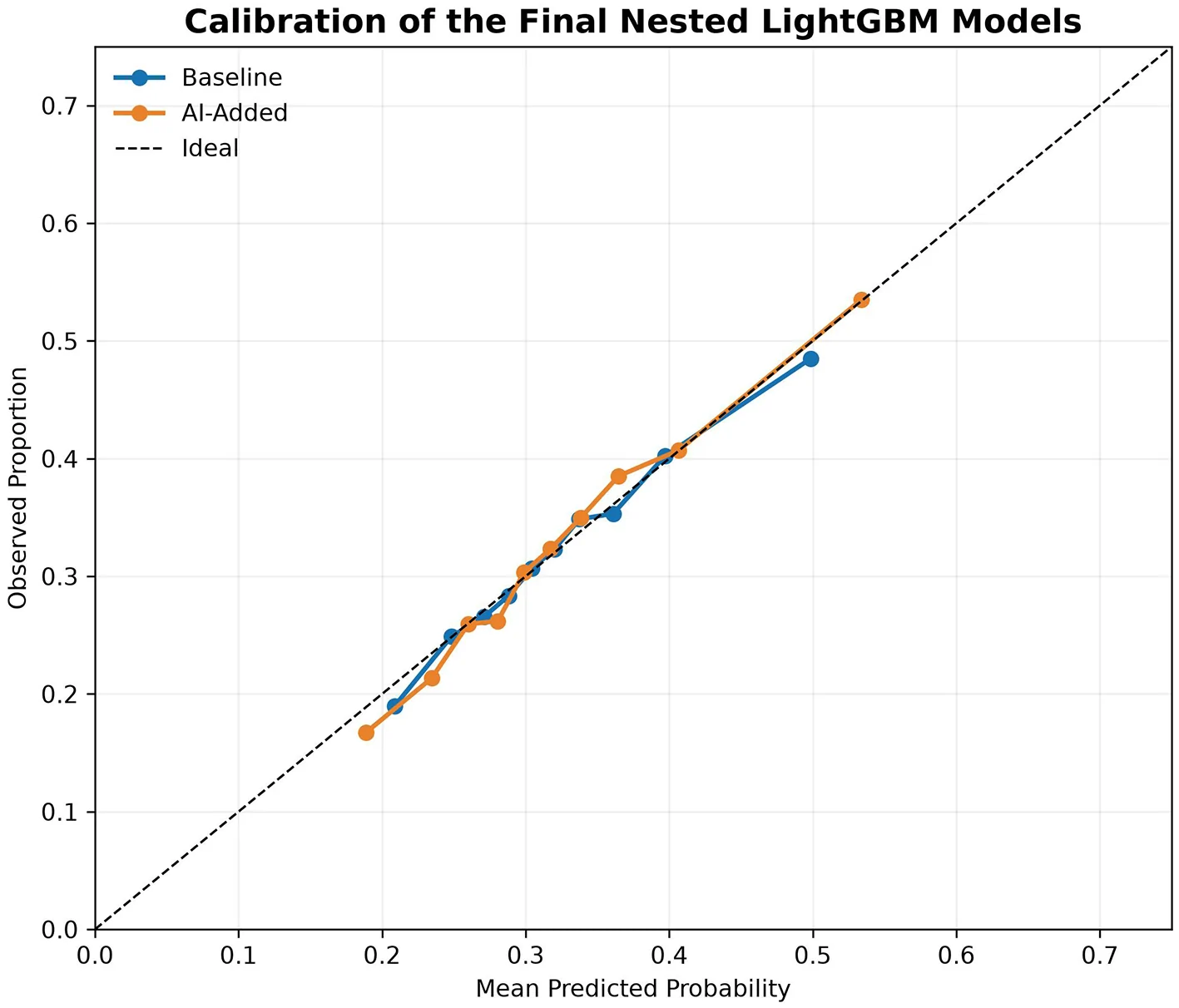
Calibration of the final nested LightGBM models. Both models were close to the diagonal reference line in the central probability range, while predicted probabilities were concentrated in a moderate range.

Figure 5 shows the calibration curves for the final matched models. Both curves were close to the diagonal reference line in the central probability range. Final Model 2 showed slightly improved probability quality, consistent with its lower Brier score and log loss, although predicted probabilities remained concentrated in a moderate range. This supports use of the model for descriptive, group-level predictive assessment rather than deterministic individual-level decisions.

**Figure 6:**
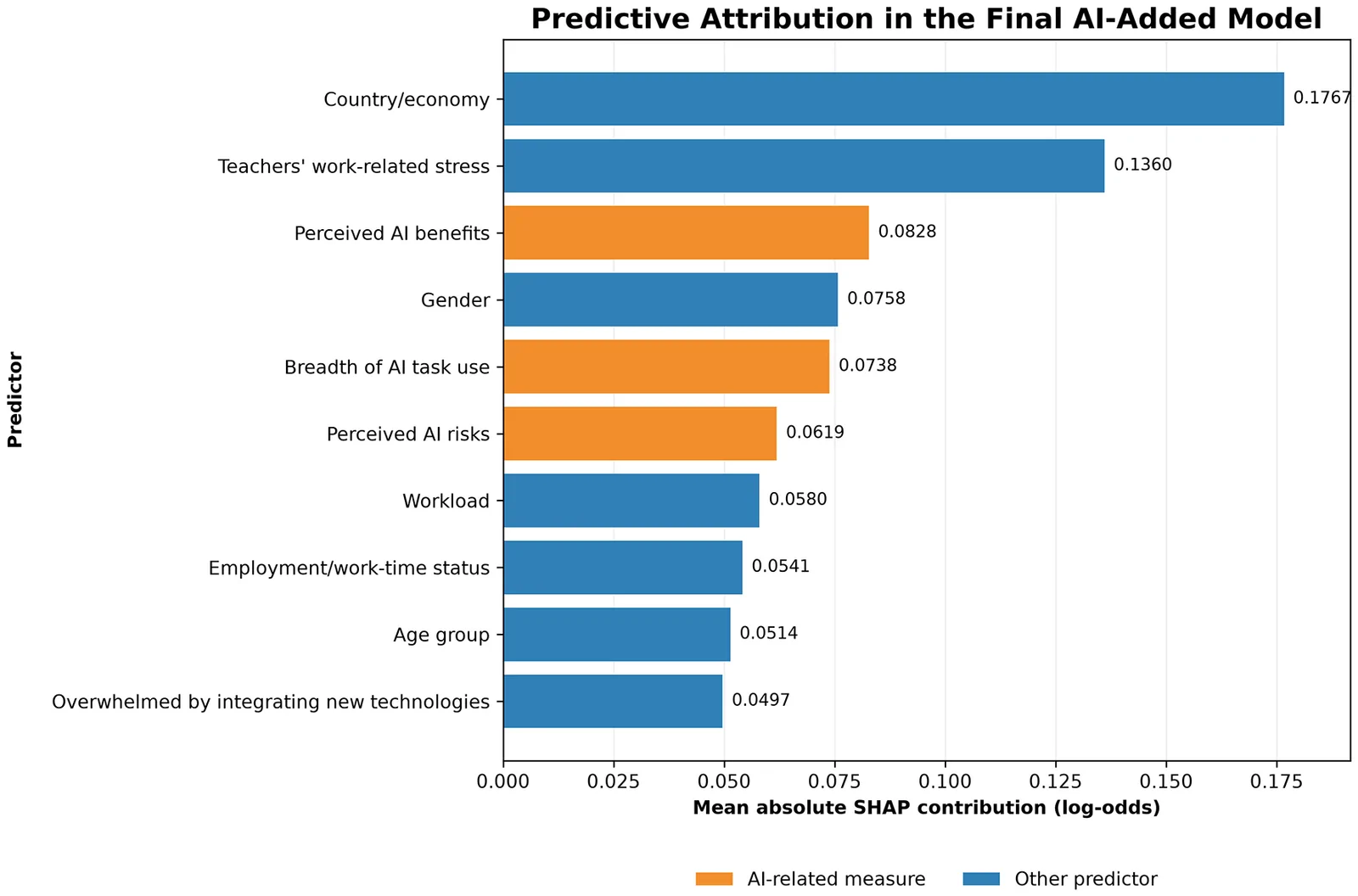
Predictive attribution in the final AI-added model. Source-variable SHAP contributions were aggregated over encoded columns and ranked by mean absolute magnitude. AI-related measures are shown in orange. SHAP values describe fitted-model attribution, not statistical significance or causal effects.

SHAP analysis ranked country/economy, the broad work-related stress index, perceived AI benefits, gender, breadth of AI task use, perceived AI risks, workload, employment/work-time status, age group, and feeling overwhelmed by integrating new technologies as the ten leading contributors to Model 2 predictions. Because predictors are correlated, ranks may redistribute importance among AI benefits, risks, task breadth, and barriers. In addition, the task-breadth attribution can partly reflect questionnaire routing through its missingness indicator rather than observed breadth alone. The results describe predictive attribution only; they do not show statistical significance, causal influence, pedagogical quality, or intervention priority. The top 10 source-level predictors are reported in Table 9 and visualized in Figure 6.

**Table 9:** Final Model 2 top predictors ranked by mean absolute SHAP value.

Rank	Predictor	Mean absolute SHAP
1	Country/economy	0.1767
2	Teachers' work-related stress	0.1360
3	Perceived AI benefits	0.0828
4	Gender	0.0758
5	Breadth of AI task use	0.0738
6	Perceived AI risks	0.0619
7	Workload	0.0580
8	Employment/work-time status	0.0541
9	Age group	0.0514
10	Overwhelmed by integrating new technologies	0.0497

The separate algorithm comparison used six conventional linear and ensemble/tree methods on the same training rows, test rows, source predictors, and country-by-school isolation rule. Hyperparameter budgets were not exhaustively equalized, so it remains a descriptive sensitivity analysis and was not used as confirmatory evidence for the AI block.

## 5 Discussion

AI-related measures supplied statistically stable but modest incremental predictive information for predicting relatively high SLAA after teacher background, country/economy, and the broad work-related stress index were included. This pattern cannot establish that AI improves teaching: teachers with stronger pre-existing practices, technological competence, resources, or positive attitudes may be more likely to use AI across more task types and perceive benefits. Moreover, because 59.1% of task-breadth values were absent largely through questionnaire routing, its source-level SHAP importance may partly reflect eligibility or questionnaire-pathway information. It cannot be interpreted as evidence about actual breadth of use alone, pedagogical maturity, or quality.

The matched nested design is important because it separates AI-block predictive information from general teacher and system-level variation. Although the direction of the AI-added difference was consistent across alternative thresholds, the continuous outcome, survey-weighted evaluation, and most country-specific test subsets, its magnitude was not invariant to weighting. The unweighted baseline and AI-added AUROCs were 0.6021 and 0.6299, whereas weighted evaluation yielded 0.5878 and 0.6345; thus, the incremental AUROC difference increased from 0.0277 to 0.0466. This sensitivity suggests substantive influence from survey weights, country-level sample composition, or unequal selection probabilities, and it limits population-level interpretation. The country results also showed substantial heterogeneity: seven countries/economies did not show a positive AUROC difference, and the direction of the risk–SLAA correlation was almost evenly split. Response styles, educational policies, AI infrastructure, working conditions, and measurement comparability differ across systems. Consequently, a pooled multinational pattern should not be translated into one uniform teacher-training strategy or AI policy.

Implications for public health are necessarily indirect. Communication, feedback, adaptation, and social-emotional support are relevant to health education and health-promoting schools, but this dataset did not measure student health literacy, behavior, clinical knowledge, or programme outcomes. The results can motivate future evaluation of responsible AI support in those settings; they cannot demonstrate benefits to public-health education.

From a methodological perspective, the study emphasizes auditability rather than novelty through an opaque model architecture. The codebook-aware cleaning step was necessary because survey-specific response codes can otherwise be misinterpreted as substantive values, especially for conditional AI-module items. The matched nested design then converted the substantive question into a direct empirical comparison: whether AI-related variables improve prediction after teacher background, country/economy, and teachers' work-related stress have already been included. This is more conservative than interpreting a single full-model variable ranking, because it requires AI predictors to demonstrate incremental predictive information under the same split, model class, and early-stopping procedure. The paired bootstrap analysis attached uncertainty intervals to the metric differences, while the SHAP analysis made fitted-model attribution inspectable.

The experiments also show why the highest AUROC alone should not determine the scientific reporting model. Random forest produced the highest comparison AUROC in both scenarios but worse Brier score and log loss than the boosted-tree models. The matched LightGBM pair was retained for the pre-specified AI-block contrast because it combined regularization, country-by-school validation, early stopping, probability-quality metrics, and inspectable SHAP attribution. This distinction is important in education and public-health-adjacent interpretation, where a model should support transparent analysis rather than become an unexamined classifier of teachers.

The SHAP results do not identify actionable factors or justify intervention priorities. Instead, perceived benefits, perceived risks, task-breadth/routing information, and the broad stress index are model-dependent signals that may be used to formulate questions for future longitudinal or intervention studies. Such studies could separately measure AI competence, frequency and quality of task use, workload, wellbeing, implementation feasibility, and independently assessed teaching or student outcomes before making recommendations for professional development or policy. Dedicated studies would also be required before drawing practice implications for health education or health-promoting schools.

### 5.1 Limitations

This study has several limitations. First, all focal variables were measured at one time point, so temporal ordering and causality cannot be established; reverse associations and common-method bias are plausible. Second, SLAA is a recent, class-specific teacher report rather than student outcomes, observations, or supervisor ratings. The upper-quartile label denotes only relative position within country/economy and ISCED stratum; it is not objectively superior teaching performance or a common cross-national standard. Third, AI-module routing and missingness reduced the raw file substantially. In particular, 59.1% missingness in task breadth was largely routing-related, so its SHAP attribution can conflate observed breadth with eligibility or questionnaire-pathway information. Train-only imputation and missingness indicators retain records for prediction but do not correct selection bias; the low observed background standardized differences do not rule out unmeasured selection. Fourth, unweighted model fitting targets the analytic sample rather than design-based TALIS populations. The direction of weighted results was similar, but baseline performance and the incremental magnitude changed meaningfully; clustering, unequal selection probabilities, country sample-size imbalance, response styles, and cultural/linguistic measurement comparability remain concerns. Fifth, the 20-item stress index covers heterogeneous sources and experiences. Its high alpha and omega may partly reflect item count, and its mean communality of 0.350 does not establish a single latent stress dimension; its use as one broad adjustment variable may obscure dimension-specific patterns. Full measurement invariance, convergent validity, and discriminant validity were not established. Sixth, the Health/PE scenario mainly represents school physical-education/health teachers, is not representative of public-health, medical, or university education, and is a same-source descriptive subset rather than external validation. The study did not measure public-health curricula or health outcomes. Seventh, modest discrimination precludes individual decision use. Finally, SHAP explains fitted predictions, can redistribute importance across correlated variables, and establishes neither statistical association nor causal or actionable effects.

## 6 Conclusions

This study examined measured AI-related variables and relatively high self-reported lesson-aim attainment using TALIS 2024 teacher data. AI-related measures provided modest incremental predictive information beyond teacher background, country/economy, and a broad work-related stress index. Perceived AI benefits, task-breadth/routing information, and perceived AI risks contributed to fitted predictions, but their SHAP ranks are neither statistical effects, causal effects, actionable factors, nor policy priorities.

Future work should extend this study using longitudinal or repeated-measure data to examine whether changes in AI use, AI literacy, and AI-related professional development are followed by changes in independently assessed teaching practices. Future studies should also combine teacher reports with student health literacy, student learning outcomes, classroom observations, or administrative indicators to reduce common-method bias. Dedicated, directly sampled studies would be needed to assess whether similar predictive patterns occur in mental health education, nutrition education, disease-prevention education, or health-promotion programmes.

In conclusion, this study provides an auditable framework for exploring AI-related predictive information in general school settings. It does not show that AI improves teaching or health outcomes and should not support teacher-level decisions. The context-dependent findings instead identify hypotheses for longitudinal, intervention, and externally validated research, including dedicated studies in health education and health-promoting schools.

## Data Availability

Publicly available data were analyzed in this study. The TALIS 2024 public-use data can be obtained from the official OECD TALIS 2024 Database (https://www.oecd.org/en/data/datasets/talis-2024-database.html). The raw respondent-level files are not redistributed by the author and should be downloaded directly from the OECD source. The analysis code, execution instructions, variable crosswalk, data-cleaning rules, random seeds, software and package metadata, model parameters, and non-identifiable derived outputs supporting the reported findings are available from the corresponding author upon reasonable request. Requests should be directed to zhufangmao@126.com.

## References

[1] Miao F Holmes W. Guidance for Generative AI in Education and Research. Paris: UNESCO. (2023).

[2] Kasneci E Sessler K Küchemann S Bannert M Dementieva D Fischer F . ChatGPT for good? On opportunities and challenges of large language models for education. Learn Indiv Differ. (2023) 103:102274. doi: 10.1016/j.lindif.2023.102274

[3] World Health Organization UNESCO. Making Every School a Health-Promoting School: Global Standards and Indicators. Geneva: World Health Organization and UNESCO. (2021).

[4] World Health Organization. Health promoting schools (n.d.). Available online at: https://www.who.int/health-topics/health-promoting-schools (Accessed June 17, 2026).

[5] Zawacki-Richter O Marín VI Bond M Gouverneur F. Systematic review of research on artificial intelligence applications in higher education-where are the educators? Int J Educ Technol High Educ. (2019) 16:39. doi: 10.1186/s41239-019-0171-0

[6] Hwang GJ Xie H Wah BW Gašević D. Vision, challenges, roles and research issues of artificial intelligence in education. Comput Educ. (2020) 1:100001. doi: 10.1016/j.caeai.2020.100001

[7] Ouyang F Jiao P. Artificial intelligence in education: the three paradigms. Comput Educ. (2021) 2:100020. doi: 10.1016/j.caeai.2021.100020

[8] Lo CK. What is the impact of ChatGPT on education? A rapid review of the literature. Educ Sci. (2023) 13:410. doi: 10.3390/educsci13040410

[9] Nguyen A Ngo HN Hong Y Dang B Nguyen BPT. Ethical principles for artificial intelligence in education. Educ Inf Technol. (2023) 28:4221–41. doi: 10.1007/s10639-022-11316-w 36254344 PMC9558020

[10] Celik I Dindar M Muukkonen H Järvelä S. The promises and challenges of artificial intelligence for teachers: a systematic review of research. TechTrends. (2022) 66:616–30. doi: 10.1007/s11528-022-00715-y

[11] Almasri F. Exploring the impact of artificial intelligence in teaching and learning of science: a systematic review of empirical research. Res Sci Educ. (2024) 54:977–97. doi: 10.1007/s11165-024-10176-3

[12] Tan X Cheng G Ling MH. Artificial intelligence in teaching and teacher professional development: a systematic review. Comput Educ. (2025) 8:100355. doi: 10.1016/j.caeai.2024.100355

[13] Miao F Cukurova M. AI Competency Framework for Teachers. Paris: UNESCO (2024).

[14] Topol EJ. High-performance medicine: the convergence of human and artificial intelligence. Nat Med. (2019) 25:44–56. doi: 10.1038/s41591-018-0300-7 30617339

[15] Rajpurkar P Chen E Banerjee O Topol EJ. AI in health and medicine. Nat Med. (2022) 28:31–8. doi: 10.1038/s41591-021-01614-0 35058619

[16] Moor M Banerjee O Abad ZSH Krumholz HM Leskovec J Topol EJ . Foundation models for generalist medical artificial intelligence. Nature. (2023) 616:259–65. doi: 10.1038/s41586-023-05881-4 37045921

[17] Singhal K Azizi S Tu T Mahdavi SS Wei J Chung HW . Large language models encode clinical knowledge. Nature. (2023) 620:172–80. doi: 10.1038/s41586-023-06291-2 37438534 PMC10396962

[18] OECD. TALIS 2024 Database (2025). Available online at: https://www.oecd.org/en/data/datasets/talis-2024-database.html (Accessed June 17, 2026).

[19] OECD. TALIS 2024 Teacher Questionnaire, ISCED 1, 2 and 3 (2024). Available online at: https://www.oecd.org/content/dam/oecd/en/about/programmes/edu/talis/talis2024questionnaires/TALIS2024TeacherQuestionnaireISCED1-2-3.pdf (Accessed June 17, 2026).

[20] OECD. Results from TALIS 2024: The State of Teaching. Paris: OECD Publishing (2025).

[21] Ke G Meng Q Finley T Wang T Chen W Ma W . LightGBM: a highly efficient gradient boosting decision tree. In: Advances in neural information processing systems. (2017). p. 3146–3154.

[22] Lundberg SM Lee SI. A unified approach to interpreting model predictions. In: Advances in neural information processing systems. (2017). p. 4765–4774.

[23] Davis FD. Perceived usefulness, perceived ease of use, and user acceptance of information technology. MIS Quart. (1989) 13:319–40. doi: 10.2307/249008

[24] Venkatesh V Morris MG Davis GB Davis FD. User acceptance of information technology: toward a unified view. MIS Quart. (2003) 27:425–78. doi: 10.2307/30036540

[25] OECD. Technical notes on analyses in this volume (2025). Available online at: https://www.oecd.org/en/publications/2025/10/results-from-talis-2024_28fbde1d/full-report/technical-notes-on-analyses-in-this-volume_286d707e.html (Accessed June 17, 2026).

[26] Breiman L. Random forests. Mach Learn. (2001) 45:5–32. doi: 10.1023/A:1010933404324

[27] Friedman JH. Greedy function approximation: a gradient boosting machine. Ann Stat. (2001) 29:1189–232. doi: 10.1214/aos/1013203451

[28] Chen T Guestrin C. XGBoost: a scalable tree boosting system. In: Proceedings of the 22nd ACM SIGKDD international conference on knowledge discovery and data mining (2016). p. 785–794. doi: 10.1145/2939672.2939785

[29] Prokhorenkova L Gusev G Vorobev A Dorogush AV Gulin A. CatBoost: unbiased boosting with categorical features. In: Advances in neural information processing systems (2018). p. 6638–6648.

[30] Grinsztajn L Oyallon E Varoquaux G. Why do tree-based models still outperform deep learning on typical tabular data? In: Advances in neural information processing systems. (2022). p. 507–520. doi: 10.52202/068431-0037

[31] Gorishniy Y Rubachev I Kartashev N Shlenskii D Kotelnikov A Babenko A. TabR: tabular deep learning meets nearest neighbors. In: International conference on learning representations. (2024).

[32] Gorishniy Y Kotelnikov A Babenko A. TabM: advancing tabular deep learning with parameter-efficient ensembling. In: International conference on learning representations. (2025).

[33] Hollmann N Müller S Purucker L Krishnakumar A Körfer M Hoo SB . Accurate predictions on small data with a tabular foundation model. Nature. (2025) 637:319–26. doi: 10.1038/s41586-024-08328-6 39780007 PMC11711098

[34] Qu J Holzmüller D Varoquaux G Le Morvan M. TabICL: a tabular foundation model for in-context learning on large data. In: Proceedings of the 42nd international conference on machine learning. PMLR (2025). p. 50817–50847.

[35] Holzmüller D Grinsztajn L Steinwart I. Better by default: strong pre-tuned MLPs and boosted trees on tabular data. In: Advances in neural information processing systems (2024). p. 26577–26658. doi: 10.52202/079017-0837

[36] Thielmann AF Kumar M Weisser C Reuter A Säfken B Samiee S. Mambular: a sequential model for tabular deep learning. arXiv preprint arXiv:2408.06291. (2024).

[37] Lundberg SM Erion G Chen H DeGrave A Prutkin JM Nair B . From local explanations to global understanding with explainable AI for trees. Nat Mach Intell. (2020) 2:56–67. doi: 10.1038/s42256-019-0138-9 32607472 PMC7326367

[38] Khosravi H Shum SB Chen G Conati C Tsai YS Kay J . Explainable artificial intelligence in education. Comput Educ. (2022) 3:100074. doi: 10.1016/j.caeai.2022.100074

[39] Brier GW. Verification of forecasts expressed in terms of probability. Monthly Weather Rev. (1950) 78:1–3. doi: 10.1175/1520-0493(1950)078 < 0001:VOFEIT>2.0.CO;2

[40] Van Calster B McLernon DJ Van Smeden M Wynants L Steyerberg EW Bossuyt P . Calibration: the Achilles heel of predictive analytics. BMC Med. (2019) 17:230. doi: 10.1186/s12916-019-1466-7 31842878 PMC6912996

[41] Efron B Tibshirani RJ. An Introduction to the Bootstrap. New York: Chapman & Hall. (1993). doi: 10.1007/978-1-4899-4541-9

[42] Pedregosa F Varoquaux G Gramfort A Michel V Thirion B Grisel O . Scikit-learn: machine learning in Python. J Mach Learn Res. (2011) 12:2825–30. Available online at: https://jmlr.org/papers/v12/pedregosa11a.html (Accessed June 17, 2026).

